# The Role of Copper in Tau-Related Pathology in Alzheimer’s Disease

**DOI:** 10.3389/fnmol.2020.572308

**Published:** 2020-09-10

**Authors:** Klara Zubčić, Patrick R. Hof, Goran Šimić, Maja Jazvinšćak Jembrek

**Affiliations:** ^1^Laboratory for Developmental Neuropathology, Department for Neuroscience, Croatian Institute for Brain Research, University of Zagreb Medical School, Zagreb, Croatia; ^2^Nash Family Department of Neuroscience, Icahn School of Medicine at Mount Sinai, New York, NY, United States; ^3^Ronald M. Loeb Center for Alzheimer’s Disease, Icahn School of Medicine at Mount Sinai, New York, NY, United States; ^4^Friedman Brain Institute, Icahn School of Medicine at Mount Sinai, New York, NY, United States; ^5^Laboratory for Protein Dynamics, Division of Molecular Medicine, Ruđer Bošković Institute, Zagreb, Croatia; ^6^Department of Psychology, Catholic University of Croatia, Zagreb, Croatia

**Keywords:** copper, tau, aggregation, oxidative stress, Alzheimer’s disease

## Abstract

All tauopathies, including Alzheimer’s disease (AD), are characterized by the intracellular accumulation of abnormal forms of tau protein in neurons and glial cells, which negatively affect microtubule stability. Under physiological conditions, tubulin-associated unit (Tau) protein is intrinsically disordered, almost without secondary structure, and is not prone to aggregation. In AD, it assembles, and forms paired helical filaments (PHFs) that further build-up neurofibrillary tangles (NFTs). Aggregates are composed of hyperphosphorylated tau protein that is more prone to aggregation. The pathology of AD is also linked to disturbed copper homeostasis, which promotes oxidative stress (OS). Copper imbalance is widely observed in AD patients. Deregulated copper ions may initiate and exacerbate tau hyperphosphorylation and formation of β-sheet-rich tau fibrils that ultimately contribute to synaptic failure, neuronal death, and cognitive decline observed in AD patients. The present review summarizes factors affecting the process of tau aggregation, conformational changes of small peptide sequences in the microtubule-binding domain required for these motifs to act as seeding sites in aggregation, and the role of copper in OS induction, tau hyperphosphorylation and tau assembly. A better understanding of the various factors that affect tau aggregation under OS conditions may reveal new targets and novel pharmacological approaches for the therapy of AD.

## Introduction

Tubulin-associated unit (Tau) is a microtubule-associated phosphoprotein predominantly expressed in neuronal cells, mainly in axons. It is essential for proper assembly, stabilization, and functioning of a microtubule network. Tau also regulates axonal transport, drives neurite outgrowth, and shapes the neuronal morphology (Choi et al., 2009; Wang and Mandelkow, 2016).

Human tau is a relatively large protein with six isoforms derived from a single gene. The isoforms range in length from 352 to 441 amino acids. Tau is divided into two functional domains: a projection domain at the N-terminus, and a microtubule-binding domain at the C-terminal part (for review, see Šimić et al., 2016; Barbier et al., 2019). Regarding tau-related pathology, the major difference between the isoforms is located in the microtubule-binding domain that contains three or four imperfect pseudorepeat units (R1–R4). Each repeat consists of a 31–32 amino acid sequence that contains a highly conserved fragment of 18 residues (octadecapeptide). Accordingly, the isoforms are termed as either 3R-tau or 4R-tau isoforms. They all encompass the third repeat R3 (residues 306–336 according to the longest human tau isoform) and differ from each other by the absence or presence of the R2 repeat. The 4R-tau isoforms bind to microtubules with higher affinity. In the healthy human brain, 3R-/4R-tau isoforms are expressed in a similar ratio, but this ratio is disturbed in Alzheimer’s disease (AD; for review, see Alavi Naini and Soussi-Yanicostas, 2015; Liu et al., 2015; Cheng and Bai, 2018). The other difference between the isoforms is the number of N-terminal inserts in the projection domain (0N, 1N, or 2N). A proline-rich region flanking the microtubule-binding domain and the projection domain facilitates tau interaction with microtubules. The N-terminal part of tau determines the spacing between microtubules and does not participate in microtubule binding (Schneider et al., 1999; Kadavath et al., 2015; Cheng and Bai, 2018; Barbier et al., 2019).

Tau binds tightly to microtubules with the small groups of evolutionarily conserved residues, whereas intervening parts stay flexible. Thus, tau remains highly dynamic despite being bound to microtubules. These small groups of microtubule-binding motifs stabilize protofilament conformation of microtubules by binding to a hydrophobic pocket at the interface between α-β-tubulin heterodimers. The mechanism is similar for 3R-tau or 4R-tau isoforms. The residues participating in binding to microtubules are critically involved in tau aggregation, a hallmark feature of AD (Kadavath et al., 2015).

## Effect of Tau Phosphorylation and Tau Kinases on Tau Function

Tau is susceptible to many posttranslational modifications that modulate its function and contribute to the heterogeneity of tau isoforms present in the brain. The diversity of biological tau functions is mainly regulated by phosphorylation that attracted much attention as a modification particularly relevant for the propagation of AD pathology (Jazvinšćak Jembrek et al., 2013; Alavi Naini and Soussi-Yanicostas, 2015; Šimić et al., 2016; Barbier et al., 2019; Miao et al., 2019). In AD tau is hyperphosphorylated and is not attached to microtubules. Hyperphosphorylation, represented as an overall increase of phosphorylation at multiple residues, reduces the binding affinity of tau for microtubules and is responsible for the loss of the physiological tau functions. Hyperphosphorylation disturbs interactions between tau and microtubules and results in microtubule instability, decreased microtubule bundling, impairment of axonal transport and neuronal architecture, and ultimately cell death (Evans et al., 2000; Alavi Naini and Soussi-Yanicostas, 2015; Barbier et al., 2019).

Besides interacting with microtubules, tau at its N-terminal part may bind signaling proteins that contain Src homology 3 (SH3) domains, including the non-receptor tyrosine kinase Fyn. Fyn is elevated in the AD brain, particularly in neurons with hyperphosphorylated tau, and contributes to the increase in tau levels by promoting tau translation. Fyn itself also may phosphorylate tau at Tyr18, and this phosphorylation step has been linked to AD pathology (Lee et al., 2004). However, the most prominent role of Fyn in AD is related to the effects of tau on Aβ toxicity. It is considered that the mislocalization of phosphorylated tau from the axons into the dendritic compartments mediates the toxic effects of Aβ by locally increasing concentration of Fyn (Li and Götz, 2017; Nygaard, 2018). In physiological conditions, tau is present in low amounts in dendrites but has an important role in sequestering Fyn to postsynaptic dendritic membranes where Fyn strengthens interactions between NMDA receptors containing NR2B subunit and postsynaptic density (PSD) protein PSD-95. This is crucial in AD for driving excitotoxic signaling cascade triggered by soluble Aβ oligomers (Alavi Naini and Soussi-Yanicostas, 2015; Nygaard, 2018).

The longest tau isoform possesses 85 residues that can be phosphorylated. Almost 45 of them are phosphorylated in the AD brain, in comparison to 10–18 from the healthy adult human brain. Very few of the phosphorylated residues occur in the N-terminal region and the microtubule-binding domain. Instead, phosphorylation sites are mainly located in the flanking regions of the microtubule-binding domain (for review, see Alavi Naini and Soussi-Yanicostas, 2015; Wang and Mandelkow, 2016). Tau phosphorylation is mediated by many kinases, at least *in vitro*. Among them, glycogen synthase kinase-3β (GSK-3β) and cyclin-dependent kinase 5 (cdk5) are considered as particularly important. Increased activity of GSK-3β, as well as upregulation of cdk5 and its activators, p35, and p25, were found in the frontal cortex of AD brain samples (Duka et al., 2013). These kinases phosphorylate tau at multiple serine and threonine residues known to be implicated in AD, and their overexpression or activation induces tauopathy-characteristic phenotypes (Terwel et al., 2008; Kremer et al., 2011; Jazvinšćak Jembrek et al., 2013; Llorens-Martín et al., 2014). GSK-3β truncated at the C-terminus is markedly increased in the AD brain, and truncation shows a positive correlation with tau hyperphosphorylation and Braak stage. Proteolytic cleavage of GSK-3β by calpain I increase its kinase activity (Feng et al., 2013; Jin et al., 2015). Both kinases are hyperactivated in oxidative stress (OS)-conditions (Shukla et al., 2012; Alavi Naini and Soussi-Yanicostas, 2015), suggesting that attenuation of ROS generation might reduce their activity and alleviate the level of tau hyperphosphorylation.

## Tau Aggregation in AD and Other Tauopathies

Besides destabilizing microtubules, hyperphosphorylated tau forms intraneuronal deposits upon detachment from microtubules. This is somewhat unexpected due to the very high solubility of tau (Schneider et al., 1999). In a self-propagating aggregation process, tau at first forms dimers and then oligomers that accumulate to form protomers. Pairs of protomers twist around each other and generate paired helical filaments (PHFs) that associate and grow into bundles of neurofibrillary tangles (NFTs; for review, see Mokhtar et al., 2013; Liu et al., 2015). As fetal tau isoform (0N3R) does not form PHF even in a highly phosphorylated state, understanding its expression and post-translational modifications may be important for future research towards the development of AD treatment and prevention (Jovanov-Milošević et al., 2012).

In addition to hyperphosphorylation, cleavage of tau by various proteases (such as caspases and calpains), is another modification important for the etiopathogenesis of AD (Kovacech and Novak, 2010; Zhou et al., 2018). Tau cleavage generates tau fragments that span the pseudorepeats of the microtubule-binding domain and are C-terminally truncated at Glu391. These truncated fragments build the structural core of PHFs. Tau fragments from the core are made of C-shaped units and are exactly three repeats in length, independent of originating from 3R- or 4R-tau isoforms (Wischik et al., 2014, 2018). Thus, the fibril core of PHFs, which is proteolytically resistant, is built by only a small part of tau, the repeat sequences. It is surrounded by a highly dynamic fuzzy coat originating from the residues from the C- and N-termini, whereas a network of intramolecular long-range electrostatic interactions links the core with this fuzzy coat (Bibow et al., 2011). Related to AD progression, truncated tau must be capable of seeding tau aggregation by triggering the conversion of full-length tau into truncated form (Wischik et al., 2014, 2018; Fitzpatrick et al., 2017). Asparagine endopeptidase, a cysteine proteinase-activated during aging and in AD, acts as an important mediator of neuropathological tau changes. It degrades tau, impairs its function in microtubule assembly, and promotes aggregation (Zhang et al., 2014b).

The whole tau protein has a low ability to aggregate *in vitro*, although fragments containing pseudorepeat units assemble much more readily based on their ability to form intermolecular disulfide bridges (Schweers et al., 1995). A prerequisite for nucleation is the formation of dimers (Friedhoff et al., 1998). Hence, in experimental conditions, PHF assembly is often promoted through the dimerization *via* disulfide bond formation and in the presence of polyanions (such as heparin) and inhibited in the presence of reducing agents that keep cysteine residues reduced, preventing the dimer formation (von Bergen et al., 2000). As for other amyloids, it is considered that β-sheet conformation is critical for the aggregation and fibril formation, although both α-helices and β-sheet structures are found in PHFs (Sadqi et al., 2002; Ma et al., 2005). However, amyloid structures are challenging for characterization as they are not entirely ordered, which complicates the precise determination of the structure-pathology relationship (Fichou et al., 2019). Nevertheless, tau aggregates appear before amyloid β (Aβ) plaques in AD, and their accumulation correlates better with the severity of AD pathology and cognitive decline than the formation of Aβ deposits (Bussière et al., 2002; Wischik et al., 2014; Šimić et al., 2017). As many years of research efforts along the Aβ cascade hypothesis did not bring improvements in AD therapy, targeting tau pathology is attracting much attention in recent days (Šimić et al., 2016; Cheng and Bai, 2018; Jadhav et al., 2019; Takeda, 2019).

The impact of other posttranslational modifications on tau function and aggregation is much less characterized. In contrast to normal tau that does not contain glycans, PHF tangles from AD brains are highly glycosylated. However, deglycosylation alone is not sufficient for tau to regain microtubule polymerization capacity, both deglycosylation and dephosphorylation are required for promoting microtubule assembly. After deglycosylation, PHFs are converted into bundles of straight filaments indicating a role of glycosylation in PHF formation (Wang et al., 1996; Takahashi et al., 1997). Another important posttranslational modification that may regulate tau function is acetylation at lysine residues. Similar to phosphorylation, acetylation inhibits tau function in microtubule assembly by impairing interaction with microtubules and promotes the formation of tau fibrils. Lys280 from the microtubule-binding domain is considered to be one of the major sites of tau acetylation. In brain tissue of Tg mouse models and in extracted PHFs form cortical regions of various human 4R or 3R/4R tauopathies, increased levels of acetylation, specifically at Lys274 and Lys280, were observed in pathologically insoluble, hyperphosphorylated tau aggregates (Cohen et al., 2011; Alavi Naini and Soussi-Yanicostas, 2015). A better understanding of the contribution of other post-translational tau modifications on tau pathology, particularly during OS, might bring some novel opportunities in AD treatment.

The human brain is neither susceptible only to neuronal tau accumulation nor are tau aggregates specific for AD. Besides, aging itself leads to the build-up of tau tangles, primarily in the medial temporal lobe, which is known as primary age-related tauopathy (Crary et al., 2014; Huang et al., 2016; Demaegd et al., 2018). In more than 30 different forms of tauopathies, inclusions of hyperphosphorylated tau with various morphological appearances can be found in neurons, astrocytes, and oligodendrocytes (Alavi Naini and Soussi-Yanicostas, 2015; Kahlson and Colodner, 2016). Of note, tau is normally expressed in glial cells, particularly in oligodendrocytes, although at a much lower level in comparison to neurons. The list of tauopathies includes, but is not limited to, frontotemporal dementia, progressive supranuclear palsy, corticobasal degeneration, argyrophilic grain disease, Gerstmann–Sträussler–Scheinker disease, white-matter tauopathy with globular glial inclusions, as well as several other forms of dementia. At the molecular level, a distinction between different tauopathies is based on phosphorylation, and the number of tau isoforms displayed in aggregates (for review, see Alavi Naini and Soussi-Yanicostas, 2015). For example, in AD all six tau isoforms are found in aggregates.

The contribution of glial tau pathology to the pathogenesis of tauopathies is not well understood, although at least theoretically, its targeting may bring some therapeutic hope for AD. Namely, it is considered that tau pathology in glial cells disrupts the normal role of glia in supporting neuronal functioning. These include the role of astrocytes in maintaining the blood-brain barrier and modulation of synaptic function, particularly through the secretion and uptake of glutamate, as well as the myelinating function of oligodendrocytes (for review, see Kahlson and Colodner, 2016; Leyns and Holtzman, 2017).

## Tau Spreading

The progressive nature of AD is largely based on the spreading of misfolded tau through synaptically interconnected neural pathways. As such, extracellular tau enters cells and then triggers the formation of tau fibrils from the intracellular tau pool. The small and soluble oligomeric tau forms are considered as the most pathogenic and transmissible moieties. Hence, the inhibition of oligomer formation and clearance of oligomeric forms of low molecular weight could represent a valuable therapeutic approach (Fichou et al., 2019). However, a prerequisite for this is understanding the aggregation process and identification of various AD-related biological factors and components of the intracellular environment that may affect its dynamic.

Small oligomers and prefibrillar species enable propagation of tau pathology through a cell-to-cell transmission manner (Lasagna-Reeves et al., 2012; Alavi Naini and Soussi-Yanicostas, 2015; Wang and Mandelkow, 2016) or *via* microglial cells (Španić et al., 2019). Tau enters at both somatodendritic and axonal compartments and proceeds anterogradely and retrogradely, thus spreading both trans-synaptically and transcellularly (Demaegd et al., 2018). The two major components of the propagation process are a templated misfolding or “seeding” (i.e., the ability of abnormal tau to induce a conformational change of the naturally folded tau protein) and cell-to-cell spreading (Demaegd et al., 2018). It is likely that tau propagates cell-to-cell through tunneling nanotubes (filamentous channels) or is released into the extracellular space in a free form or through transport vesicles by various secretion mechanisms, and is then subsequently taken up by nearby cells (Demaegd et al., 2018). However, a more recent epidemic spreading model predicts the spreading of tau from cell to cell only through neuronal projections, not the extracellular space (Vogel et al., 2020). Tau also spreads ubiquitously through the communication pathways during normal aging, particularly in the medial temporal cortex. In AD, the process is facilitated in regions with Aβ-burden and results in the spreading of tau outside from the medial temporal lobes into the nearby neocortex, in a good correlation with the level of neurodegeneration and cognitive impairment. NFTs are first formed in the brainstem and transentorhinal cortex from where they outspread to the anterior hippocampus, temporal association neocortex, and ultimately primary sensory cortex through secondary seeding events (Šimić et al., 1998, 2009; Vogel et al., 2020).

There is evidence in support of cell-to-cell tau spreading, a phenomenon that is hard to prove by examining the post-mortem human brain. In non-neuronal cells overexpressing tau, hyperphosphorylation accompanied by caspase-3-mediated cleavage at Asp421 promoted extracellular excretion of tau (Plouffe et al., 2012). A spread of pathological tau in a cell type-specific manner was demonstrated after the intracerebral injection of AD brain extract enriched in pathological tau into the hippocampus and overlying cortex of human mutant P301S tau transgenic mice (PS19 mice; Boluda et al., 2015). Similarly, injection of oligomeric and hyperphosphorylated tau from AD brain (AD P-tau) into the hippocampus of human tau transgenic mice resulted in tau aggregation pathology, as well as tau hyperphosphorylation, in the contralateral hippocampus and ipsilateral cortex (Miao et al., 2019). Injection of brain lysate containing tau aggregates into the normal brain or the brain of young transgenic mice may result in severe tau pathology at the injection site, and in anatomically connected regions (Leyns and Holtzman, 2017), suggesting that misfolded tau protein propagates to neighboring cells along synaptically connected circuits spatially and temporally (Lasagna-Reeves et al., 2012; Liu et al., 2012).

By searching for the minimal sequence capable of defining the self-propagating process, it was found that a peptide spanning the full R3 repeat may form amyloid fibril and in a self-propagating manner, induce misfolding of the microtubule-binding domain and spread the disease (Stöhr et al., 2017).

## Conformational Changes Required for Tau Polymerization

It is generally accepted that tau hyperphosphorylation promotes detachment from microtubules and its polymerization and aggregation. PHFs can be formed from the fragments of tau protein that lack a majority of the phosphorylation sites (Wille et al., 1992). Also, PHFs isolated without protease digestion are labeled by antibodies directed against phosphorylated epitopes in the N-terminal part of tau, but after removal of a fuzzy coat, the immunoreactivity is lost. This indicates that a fuzzy coat contains most of the amino acid residues that are phosphorylated (Šimić et al., 2016; Wischik et al., 2018). Moreover, tau phosphorylation at specific phosphorylation sites may antagonize PHF formation. Thus, phosphorylation at Ser214 and Ser262 prevents tau binding to microtubules and PHFs assembly, suggesting that phosphorylation has a protective role by preventing tau aggregation (Schneider et al., 1999). In another study, the formation of NFTs was associated with reduced oxidative damage, leading to the conclusion that tau phosphorylation could have an antioxidative function, serving as a response intended to maximally protect neuronal structure and function against oxidative injury (Smith et al., 2002; Su et al., 2010). As the self-assembling of the constructs corresponding to the repeat region occurs after the formation of cross-linked dimers, it is suggested that an increase in neuronal redox potential and excessive protein oxidation are mainly responsible for PHFs formation (Wille et al., 1992; Schweers et al., 1995).

Therefore, at the root of tau oligomerization are conformational changes that make tau more prone to aggregation. The aggregation is initiated at the nucleation sites through the local transition from a random coil to a β-sheet structure. As mentioned, dimerization *via* disulfide bond formation (cross-linking) is essential for nucleation, but many conformational and structural rearrangements are required for the transition from dimers and early oligomeric forms to mature fibrils (Friedhoff et al., 1998; von Bergen et al., 2005; Ganguly et al., 2015). Both α-helices and β-sheet structures are found in *ex vivo* PHFs (Ma et al., 2005). Some conformational changes may be induced by tau phosphorylation, although, as already mentioned, low phosphorylated tau is also able to polymerize. Hence, it is hypothesized that some other mechanisms, such as OS, act synergistically with phosphorylation, and induce conformational changes required for PHF assembly (Pérez et al., 2000).

## The Role of Hexapeptide Motifs in Tau Aggregation

The PHF core being predominantly built from the repeat domains (Tomoo et al., 2005; von Bergen et al., 2005; Wischik et al., 2018), the microtubule-binding domain plays an important role in tau aggregation. The R3 repeat unit present in all tau isoforms contains the sequence that may act as the nucleation core critical for the initiation of tau oligomerization (von Bergen et al., 2000; Ganguly et al., 2015; Stöhr et al., 2017). Identification of the smallest tau elements capable of nucleation is important as could represent conformational targets for therapeutic interventions by specifically designed inhibitors aimed to prevent tau aggregation. von Bergen et al. (2000) have identified a fragment containing R3 repeat plus some residues from the flanking region as the shortest peptide sequence with a secondary structure element capable to initiate tau aggregation into PHFs. The fragment originated from the fetal tau isoform and was named PHF43. PHF43 self-assembles quickly into the straight thin filaments. Although the periodic supertwist characteristic for PHFs found in AD was absent from the filaments formed, seeds derived from these fibers nucleated the formation of PHFs from the larger tau fragments and the full-length tau. PHFs assembly was accompanied by an increase in the β-structure content (von Bergen et al., 2000). Within PHF43, based on the highest potential for β-structure formation, a hexapeptide ^306^VQIVYK^311^ (PHF6) was identified as a minimal interaction motif that underlies interactions between tau or tau-derived fragments. The R2 repeat contains a similar nucleating motif (PHF6*, ^275^VQIINK^280^) that also includes residues with a high propensity for β conformation, but is present only in isoforms with four repeats (von Bergen et al., 2001). Other studies confirmed the critical involvement of PHF6 or PHF6* motifs in mediating intermolecular interactions between tau molecules and the formation of tau oligomers, suggesting that two small hexapeptide motifs are both essential and sufficient for polymerization (Goux et al., 2004; Peterson et al., 2008).

## PHF Assembly

PHF assembly is probably triggered by these hexapeptide fragments that locally form β-structure embedded in a random-coil structure of the whole protein. Dimerization at cysteine residues may bring hexapeptide motifs into close vicinity, facilitating interactions between β-sheets (von Bergen et al., 2000). Accordingly, mutation of cysteine residues in the R3 sequence changed amyloid morphology and their physical properties and reduced seeding ability (Stöhr et al., 2017). Similarly, if any residue from the PHF6 or PHF6* were replaced with proline, which is a β-sheet disrupting residue, the ability of polymerization would be prevented (von Bergen et al., 2001). von Bergen et al. (2000) also reported that only a small part of the entire protein, with a high β-sheet-forming propensity, serves as a seed for assembly, whereas C- and N-terminal tails protrude from the ordered core forming a fuzzy coat. However, further studies have revealed that in addition to PHF6 and PHF6*, other tau-originating peptides from the microtubule-binding domain and with the high amyloidogenic predispositions, also may act as primary nucleating sequences and promote the formation of various amyloids, such as filaments, tubes, ribbons or rolled sheets (Moore et al., 2011).

**Figure 1:**
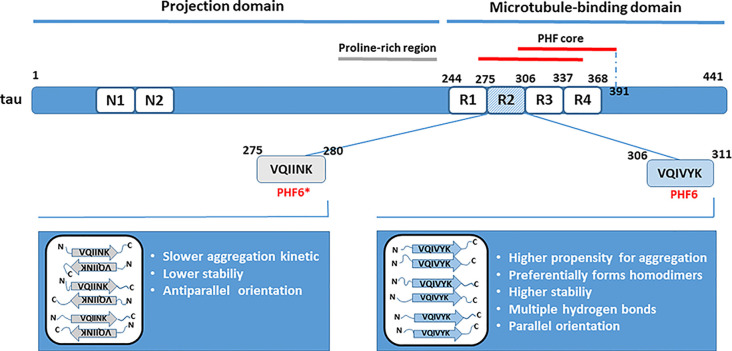
Role of hexapeptide motifs PHF6 and PHF6* in tau dimerization. Residues from the microtubule-binding domain are critically involved in tau aggregation. Studies *in vitro* have revealed that the nucleating sites from the R2 (PHF6*) and R3 (PHF6) repeat units seed tau aggregation. The PHF6 motif is a predominant trigger of tau dimerization based on its intrinsically higher propensity for aggregation, higher stability due to hydrogen bonding, and parallel orientation of peptide sequences in dimers. The positions of amino acid residues forming the paired helical filaments (PHFs) core are indicated according to Wischik et al. (2018).

In larger tau fragments, PHF6 and PHF6* may interact with each other and with themselves, generating a heterogeneous mixture of oligomeric species as a result of intermolecular interactions (PHF6-PHF6, PHF6-PHF6* and PHF*-PHF*; Peterson et al., 2008; Ganguly et al., 2015). Nevertheless, a small peptide fragment containing PHF6 (R3/wt) exhibited a higher propensity for aggregation than fragment containing PHF6*(R2/wt), and R3/wt assembly was delayed in the presence of R2/wt (Ganguly et al., 2015). R3/wt peptide preferentially formed homodimers, although stable R3/wt-R2/wt heterodimers were also present and were more stable than R2/wt homodimers. Individual chains within the R3/wt homodimers preferred a parallel orientation, whereas dimers consisting of R2/wt peptides adopted an antiparallel conformation that is in general indicative of a slower aggregation kinetics. In parallel alignment, it is easier for β-sheets to form a steric zipper that is critical for the formation of fibrillary aggregates. Besides, multiple hydrogen bonds were formed between the R3/wt peptides in more than 95% of all dimers formed, and they conferred to the stability of dimers. For comparison, in R2/wt dimers, only 10–20% of them had four or more hydrogen bonds (Figure 1). Taken together, it was concluded that the role of PHF6 as a seed that drives tau aggregation seems dominant over the role of PHF6* (Ganguly et al., 2015). However, inhibitors based on the structure of PHF6 were not effective in preventing seeding by full-length tau, and a recent study suggested that PHF6* could be the more potent initiator of tau aggregation and seeding (Seidler et al., 2018).

Nevertheless, further studies have questioned whether hexapeptide sequences alone represent the self-propagating core, and continued the search for the minimal fragment capable of inducing template misfolding and aggregation. It was found that PHF6 formed amyloids very rapidly, but gradual elongation in five-residue steps to the full length of the R3 repeat slowed down the amyloid formation and markedly altered fiber morphology (Stöhr et al., 2017). More importantly, by studying externally induced aggregation of the endogenously expressed tau244–372 fragment with the disease-associated mutations P301L/V337M [named tau-RD(LM)], a large difference of these growing-in-length peptides was observed in their ability to induce aggregation. Amyloids formed from the 31-residue long R3 sequence were as effective as fibrillized tau-RD in inducing tau-RD(LM) aggregation. On the contrary, PHF6 and growing peptides shorter than 20 residues, were not able to seed aggregation of tau-RD(LM). Thus, in extension from hexapeptide to peptide containing 31 residues, the resulting peptides formed amyloids more slowly but at the same time were more potent to induce template misfolding (Stöhr et al., 2017).

Despite the great efforts that have been put in untangling the process of tau aggregation *in vitro*, there are still many open questions. It must be taken in mind that these experiments were performed on arbitrary tau fragments, largely in the absence of post-translational modifications. Hence, it is still not clear if the arrangement of recombinant filaments resembles those present in a real biological system, where many factors may affect tau assembly, particularly in the diseased brain (Fichou et al., 2019). To understand the assembling process *in vivo* better, cryoelectron microscopy has been employed for monitoring PHFs and straight filaments from the AD brain. The analysis revealed that they consist of hyperphosphorylated, full-length tau and can nucleate aggregation of human full-length tau in cultured cells. Even though *in vitro* studies demonstrated higher propensity of R2 and R3 pseudorepeats for aggregation, the ordered core of both types of filaments obtained from AD brain was made of pairs of identical protofilaments containing C-shaped subunits from residues 306 to 378, encompassing the entire R3 and R4 repeat units plus 10 residues following, while N- and C-termini formed disordered fuzzy coat as already explained (Fitzpatrick et al., 2017). Importantly, it seems that the structures of tau filaments are similar among AD patients indicating a common pattern of tau folding (Falcon et al., 2018). Hence, further studies must be directed towards a better understanding of molecular mechanisms of tauopathies by examining tau filaments from the AD brain, which hopefully will improve diagnostics and accelerate the development of disease-modifying therapies.

## Oxidative Stress and Tau Aggregation

OS includes conditions in which an increase in the level of reactive oxygen and nitrogen species (ROS and RNS) surpasses the endogenous mechanisms of antioxidant defense provided by various enzymatic and non-enzymatic antioxidants. Through the increased formation of ROS and RNS, OS induces oxidative damage of essential macromolecules that threatens neuronal structure and ultimately function (Nunomura et al., 2001; Alavi Naini and Soussi-Yanicostas, 2015; Jazvinšćak Jembrek et al., 2015; Liu et al., 2015, 2017).

**Figure 2:**
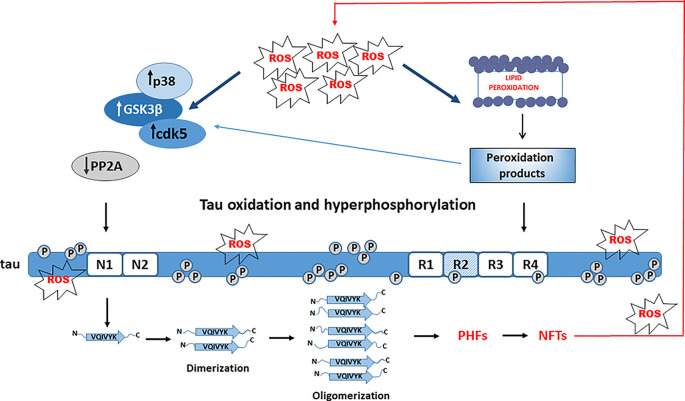
Role of tau hyperphosphorylation and oxidation in tau aggregation. Oxidative stress (OS) activates Alzheimer’s disease (AD)-related kinases (GSK-3β, cdk5, and p38) and inhibits tau dephosphorylation by PP2A, leading to tau hyperphosphorylation. OS-mediated lipid peroxidation results in toxic aldehydes that further activate tau kinases and trigger conformational changes of hyperphosphorylated tau promoting aggregation.

When compared to other cell types, neurons are particularly sensitive to OS and ROS- and RNS-mediated injury (Cobley et al., 2018; Wang et al., 2020). Many lines of evidence indicate that OS is an important early event in the pathogenesis of AD neurodegeneration (Nunomura et al., 2001; Mondragón-Rodríguez et al., 2013; Alavi Naini and Soussi-Yanicostas, 2015; Liu et al., 2015, 2017; Wang et al., 2020; Yeung et al., 2020). Acute OS may differentially affect tau phosphorylation, but chronic exposure to OS promotes tau hyperphosphorylation and makes tau more prone to oligomerization and formation of NFTs (Patil and Chan, 2005; Su et al., 2010; Mondragón-Rodríguez et al., 2013; Liu et al., 2015; Jazvinšćak Jembrek et al., 2018). Thus, prolonged OS induced by sub-lethal inhibition of glutathione synthesis increased phosphorylation at PHF-1 epitope in differentiated M17 neuroblastoma cells, probably due to increased activity of kinases JNK and p38 and reduced activity of protein phosphatase 2A (PP2A). Of note, activation of JNK and p38 was observed *in vivo*, together with colocalization to NFTs (Su et al., 2010). As the phosphorylation at the PHF-1 site is required for the formation of tau filaments, these results are also indicative of tau aggregation that was confirmed by the presence of high molecular weight bands (Su et al., 2010). Based on these findings it is suggested that OS precedes tau hyperphosphorylation and aggregation (Figure 2). In HT22 neuronal cells, OS alone was not capable to increase tau phosphorylation but changed the pattern of tau phosphorylation. When OS was combined with the inhibition of phosphatase activity, tau was highly phosphorylated and also a poor substrate for proteasomal degradation, indicating that both oxidation and hyperphosphorylation of tau are important for tau aggregation (Poppek et al., 2006).

Acrolein, a peroxidation product from arachidonic acid that is increased in the AD brain, also promotes tau hyperphosphorylation by activating GSK-3β and p38, emphasizing again an important role of OS and lipid peroxidation in tau phosphorylation (Gómez-Ramos et al., 2003). 4-hydroxy-2-nonenal (HNE), an end-product of lipid peroxidation, increased the assembling capacity of phosphorylated 4R-tau (Pérez et al., 2000). Phosphorylated tau was more vulnerable to conformational changes in reaction with HNE that further accelerated its polymerization and formation of NFTs, suggesting an important role of HNE in NFTs formation (Liu et al., 2005). Hence, possibly the oxidation of fatty acids could be a prerequisite for promoting tau hyperphosphorylation, serving as a link between OS and tau filament formation (Gamblin et al., 2000). Interestingly, neuronal exposure to stearic and palmitic fatty acids does not increase tau phosphorylation. Instead, conditioned media from astrocytes treated with saturated fatty acids increased tau phosphorylation at AD-specific sites. As the effect was reduced by treatment with an antioxidant, it was concluded that astroglia-mediated OS significantly contributes to tau phosphorylation (Patil and Chan, 2005).

## Tau in an Oxidative Environment

Tau is susceptible to OS and OS is considered as an important factor contributing to NFT formation (for review, see Liu et al., 2015). NFTs are identified as sites of catalytic redox reactivity in the hippocampal tissue of AD patients (Sayre et al., 2000). Accumulation of hyperphosphorylated tau species stimulates the production of ROS and induction of OS conditions, but OS in turn directly promotes tau hyperphosphorylation. In this vicious cycle levels of both ROS and abnormal tau increase progressively, ultimately leading to neuronal death (Alavi Naini and Soussi-Yanicostas, 2015). Moreover, OS-mediated abnormal tau phosphorylation is likely a critical deregulator of physiological tau function at the synaptic terminals and an important underlying mechanism of a synaptic failure, in addition to its role in Fyn-mediated and Aβ-driven synaptotoxicity *via* excitotoxic pathways (Mondragón-Rodríguez et al., 2013).

Besides affecting tau function, OS also modulates the expression and activity of enzymes that are involved in tau phosphorylation. In neuronally differentiated PC12 cells exposed to H_2_O_2_, low concentrations of GSK-3β inhibitor offered protection against OS, whereas higher concentrations exhibited the opposite effect (Lee et al., 2007). 1,2-diacetylbenzene, a neurotoxic metabolite of an organic solvent 1,2-diethyl benzene, also promoted the production of ROS, stimulated the activity of GSK-3β, and increased phosphorylation of tau in the hippocampus of male C57BL/6 mice (Kang et al., 2017). In the hippocampus and cortex from the AD human brain, activation and distribution of p38 kinase, which is known to be induced by OS, exclusively colocalized with the intracellular neurofibrillary pathology (Zhu et al., 2000). It was demonstrated, both *in vitro* and *in vivo*, that the major consequence of Aβ-induced OS is the activation of p38 that further leads to tau hyperphosphorylation. This finding is also important as it links Aβ and tau pathology (Giraldo et al., 2014). Furthermore, in primary cortical neurons, Aβ-induced OS upregulated levels of a regulator of calcineurin (RCAN1; which inhibits the activity of the Ser/Thr tau phosphatase calcineurin), but also increased levels of GSK-3β and tau phosphorylation (Lloret et al., 2011). Similarly, neurons from RCAN1^−/−^ mice were more resistant to various oxidative challenges (Porta et al., 2007).

PP2A is the primary tau phosphatase in the brain and its activity is suppressed in the brain of AD patients (Taleski and Sontag, 2018). Inactivation of PP2A, together with the activation of GSK-3β and increased levels of malondialdehyde, an indicator of lipid peroxidation, were observed in hypoxia-induced tau phosphorylation in the rat hippocampus (Zhang et al., 2014a). Also, neuronal protein peptidyl-prolyl isomerase (Pin1) that stimulates tau dephosphorylation was found downregulated and oxidatively modified, and exhibited reduced activity in the AD hippocampus (Sultana et al., 2006). By changing *cis* to *trans* conformation of the peptide bond and vice versa, Pin1 may cause significant structural modifications and affect the development of tau pathology (Sultana et al., 2006; Kimura et al., 2013). Interestingly, in cultured cortical neurons obtained from a transgenic rat model that expresses a human truncated tau protein analogous to a variant form derived from sporadic AD, the expression of a human truncated tau protein depleted the number of mitochondria and increased levels of ROS, making neuronal cells more sensitive to various OS inducers. The authors suggested that tau truncation precedes OS in the AD and that OS could be the consequence, rather than the trigger, of tau pathology in AD (Cente et al., 2006).

OS-related factors that contribute to abnormal tau hyperphosphorylation also disturb mitochondrial distribution and induce mitochondrial dysfunction, mitochondrial OS, and deficiency of antioxidant enzymes such as mitochondrial superoxide dismutase (Melov et al., 2007; Cheng and Bai, 2018). Mitochondrial deficits in turn facilitate the generation of oxidants and create a more severe oxidative environment (Prentice et al., 2015). The induced OS also increases mitochondrial susceptibility to various stressors, including Aβ (Pérez et al., 2018), whereas Aβ enhances the production of ROS, induces lipid peroxidation, disturbs redox balance, and leads to mitochondrial dysfunction (Diana et al., 2008; Hu and Li, 2016). Hence, a better understanding of the inducers and consequences of oxidative damage on tau and neuronal functions, and the interplay between oxidative environment and tau oxidation and hyperphosphorylation in AD progression may be important for future directions in the development of novel therapeutic options based on antioxidative agents. Although outcomes of antioxidative therapy are still inconsistent, the appearance of OS early in the disease progression suggests that antioxidative agents could be efficient disease-modifying options.

## Copper as an OS-Inducer in AD

Increase in OS could be generated through the deregulation of metal homeostasis (Sayre et al., 2000), and there is evidence that metal-induced OS promotes Aβ deposition and tau pathology in AD (Greenough et al., 2013; Birla et al., 2020; Wang et al., 2020). At least partially, OS in AD has been linked to the impairment of copper homeostasis. Copper is an essential metal for the maintenance of brain function. It is an integral part of various enzymes and structural proteins with many important biochemical and physiological roles (Stern et al., 2007). It is required for myelin formation and preservation, the activity of cytochrome c oxidase in cellular respiration, proper functioning of the superoxide scavenging enzyme copper/zinc superoxide dismutase (Cu,Zn-SOD), and catecholamine biosynthesis (Gaetke et al., 2014). On the other hand, copper is a highly reactive transition metal that participates in electron-transfer reactions inside the cell. When copper is not bound, transitions between oxidized (cupric, Cu^2+^) and reduced (cuprous, Cu^+^) states may initiate redox cycling and formation of dangerous ROS. In physiological conditions concentrations of copper are very tightly regulated (Gaetke et al., 2014). Many mechanisms related to uptake, intracellular trafficking, protein binding, and disposal are engaged to ensure adequate copper supply to target proteins and keep copper ions in a bound form, preventing redox reactions and ROS formation. About 95% of copper from human plasma is bound to ceruloplasmin, and the rest to albumin, amino acids, and some complexes that may cross the blood-brain barrier (Squitti et al., 2008; Šimić et al., 2019). Accordingly, cytoplasmic concentrations of unbound copper are very low (Rae et al., 1999; Kaplan and Lutsenko, 2009; Gaetke et al., 2014). However, due to many reasons (such as environmental pollution, excess dietary intake, inborn errors of copper metabolism and some specific medical conditions), high amounts of copper may overwhelm the endogenous capacity of copper-binding and promote OS conditions (Jomova and Valko, 2011; Eskici and Axelsen, 2012; Gaetke et al., 2014; Hsu et al., 2018).

In general, monovalent copper is more abundant in healthy neurons where it occurs at very low concentrations, whereas levels of Cu^2+^ are elevated in pathological conditions and greatly contribute to its toxic effects (Bagheri et al., 2018; Kardos et al., 2018; Ahmadi et al., 2019). In the presence of endogenous antioxidants (such as glutathione and ascorbate), Cu^2+^ may be reduced to Cu^+^. Cuprous ions further initiate the formation of hydroxyl radicals through the decomposition of hydrogen peroxide (Fenton reaction). Hydroxyl radicals generated through this prooxidant reaction can form protein radicals in a reaction with amino-bearing carbons, and lipid radicals by attacking unsaturated fatty acids. Moreover, peroxynitrite formed in the reaction between superoxide and nitric oxide promotes the release of copper ions from protein complexes enabling the generation of more ROS (Zielonka et al., 2010). Hence, ROS and RNS ultimately induce protein oxidation, peroxidative damage of neuronal membranes, and DNA damage, promoting the development of the disease (Gaetke et al., 2014). Also, the interaction between Cu^2+^ ions and GSH may result in the disulfide bond formation and reduction of Cu^2+^ to Cu^+^. Although generated Cu^+^ may bind to GSH, the formation of Cu(I)-[GSH]_2_ complex generates superoxide anions by reducing molecular oxygen (Speisky et al., 2009). Taken together, excess levels of copper may exert neurotoxic effects and jeopardize neuronal functioning and viability by inducing OS conditions (Reddy et al., 2008; Jazvinšćak Jembrek et al., 2014; Sebio et al., 2019).

## Copper Dyshomeostasis in AD

The level of copper in AD is somehow controversial as both copper deficiency and copper overload were observed when analyzing serum, plasma, cerebrospinal fluid, and samples from AD brain regions (Ventriglia et al., 2012; Pu et al., 2017; Xu et al., 2017; Bagheri et al., 2018; Wang et al., 2020). For example, Pu et al. (2017) have found elevated levels of serum copper in patients with moderate and severe AD. On the contrary, post-mortem levels of copper analyzed by inductively coupled plasma mass spectrometry (ICP-MS) were reduced in all AD brain regions examined, including the entorhinal cortex and hippocampus (Xu et al., 2017). Furthermore, increased concentration of free copper, i.e., copper not bound to ceruloplasmin, together with ceruloplasmin fragmentation was observed in AD patients in a good correlation with pathological changes and functional impairment (Squitti et al., 2008). A recent meta-analysis studies revealed that total copper and unbound copper are elevated in the serum/plasma of AD patients but decreased in the brain of AD patients (Bagheri et al., 2018). Taken together, it seems that the aberrant copper homeostasis could be contributing significantly to AD pathology. Large population studies further revealed that levels of copper are negatively correlated with cognitive decline, subjects with lower blood copper have better short-term and long-term memory, and incidence of AD is higher in geographic areas with increased copper environmental concentrations (Squitti et al., 2016).

Copper ions are transported only in their monovalent (reduced) state (Macreadie, 2008). However, the imbalance of copper homeostasis changes properties of copper-binding, shifting tightly bound copper to copper being almost free in the cytoplasm. Hence, it has been suggested that decrease in the level of copper bound to proteins and increase of a loosely bound copper or free copper could explain increased copper levels observed in serum and plasma of AD patients (Squitti et al., 2008; Ventriglia et al., 2012; Bagheri et al., 2018). As specific genes participating in copper metabolism are also associated with AD development, copper dysmetabolism and the resulting increase of labile copper in affected brain areas in AD could be the most relevant factor when considering the copper contribution to AD pathological processes (James et al., 2012; Squitti et al., 2016; Bagheri et al., 2018).

In addition to copper dyshomeostasis, perturbations of the metabolism of other metal ions, including aluminum, zinc, and iron, have been reported in AD (Hegde et al., 2009; Kanti Das et al., 2015; Rana and Sharma, 2019; Wang et al., 2020). When compared to Al^3+^, Zn^2+^, and Fe^3+^, tau has the highest binding affinity for Cu^2+^ (Rane et al., 2020). Although all these metal ions are capable of oxygen-transfer that results in ROS generation, copper and iron are particularly efficient as ROS-catalysts. However, iron is present in a relatively low amount in the cerebral cortex, and its interactions with Aβ are considered unlikely. On the other hand, the reasons to focus on copper are the facts that copper is enriched in the hippocampus, Aβ-copper complexes are an important source of ROS and OS in AD, and copper is a cofactor of many enzymes essential for brain function (Kanti Das et al., 2015; Esmieu et al., 2019; Wang et al., 2020). Hence, deciphering the role of copper in AD progression, as well as better understanding of copper-mediated pathological events, will be beneficial in the context of developing novel therapeutic approaches for AD.

## Interactions Between Tau and Copper—Impact on Tau Aggregation

Various models of toxicity suggest that the gain-of-toxic function of Aβ following Cu^2+^ binding is one of the most important mechanisms of copper toxicity in AD. Copper removal attenuates aggregation of Aβ *in vitro*, promotes degradation of Aβ deposits, and prevents ROS production that is induced by the presence of Aβ/Cu^2+^ complex (Bagheri et al., 2018). The role of copper in tau aggregation and tau-mediated toxicity is not well understood. As explained before, pseudorepeats from the microtubule-binding domain have an essential role in tau aggregation, but they are also capable of copper coordination. Hence, it was suggested that copper could be involved in tau aggregation. There is evidence that copper promotes tau aggregation (von Bergen et al., 2000; Soragni et al., 2008; Kitazawa et al., 2009; Kim et al., 2018), and increased amounts of copper have been detected in NFTs (Sayre et al., 2000). In a double transgenic mouse model expressing wild-type human tau, exposure to high amounts of copper has increased tau hyperphosphorylation and accumulation in the absence of Aβ, together with the impairment of spatial learning and memory (Voss et al., 2014). Hence, this study demonstrates that an increase in tau phosphorylation is not dependent on the presence of Aβ pathology and emphasizes that targeting copper levels could be a beneficial strategy in modifying the progression of tau pathology.

Tau possesses one or more binding sites for copper, but binding affinity is not as strong as usually seen for other metalloproteins. The coordination environment allows for interactions with intracellular oxidants and reductants, and these interactions may locally increase the concentration of copper (Sayre et al., 2000). Copper coordination complexes are capable to act as inducers of catalytic redox activity in NFTs. It was shown that copper may catalyze H_2_O_2_-dependent oxidation of a reducing substrate at NFTs (Sayre et al., 2000). Furthermore, a fragment of tau protein, an octadecapeptide from the R2 unit, may reduce copper and generate H_2_O_2_ and very likely some other ROS moieties as well. During Cu^2+^ reduction, R2 is oxidized and forms dimers linked by a disulfide bond (Su et al., 2007). As explained above, these dimers are considered to be building blocks of PHFs. Interestingly, iron, another redox-active metal ion, does not catalyze tau oxidation (Su et al., 2007). This finding demonstrates that tau with inappropriately bound copper may initiate ROS formation and OS conditions. More importantly, by catalyzing tau oxidation and dimer formation after conformational tau transition, copper may promote PHF assembly and disease progression (Su et al., 2007).

## Copper Binding to R1-R4 Repeat Units From the Microtubule-Binding Domain

**Figure 3:**
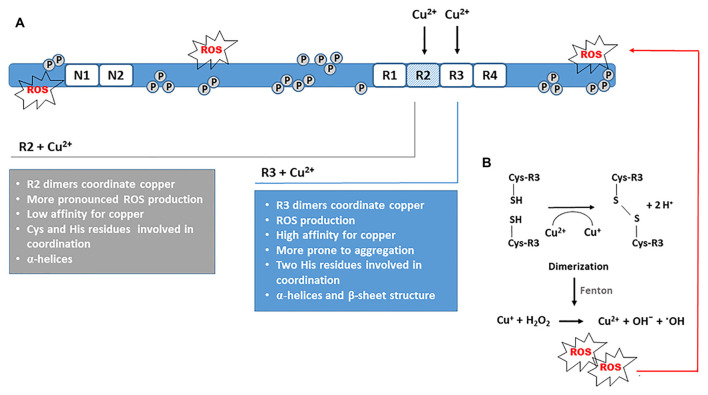
Catalytic role of Cu^2+^ in disulfide bond formation and dimerization. **(A)** *In vitro* aggregation of tau fragments derived from the R2 and R3 repeat units demonstrates the ability of copper to stimulate the formation of PHF-like fibrils. Aggregation is promoted in OS conditions. **(B)** Dimerization *via* disulfide bond formation between Cys residues is the critical initiating step that triggers tau oligomerization and further formation of fibrillar forms. Concomitantly, Cu^2+^ is reduced to Cu^+^, which catalyzes Fenton reaction and production of hydroxyl radicals. The ROS generated induces additional oxidative damage of tau protein promoting its polymerization.

The interactions between copper and individual R1-R4 repeat units or their smaller fragments involve direct binding of copper to tau-derived peptides suggesting that copper binding can induce conformational changes of the microtubule-binding domain (Ma et al., 2005; Zhou et al., 2007). In the presence of divalent copper, an increase in α-helix structure content and β-sheets represents a common spatial change. In R2 and R3 repeats other conformational and structural changes that precede, and further trigger the process of aggregation, have been observed as well. All four pseudorepeats formed aggregates after 3 days of incubation with Cu^2+^ solution. Amorphous aggregates were observed with R1 and R4 repeats, whereas Cu^2+^ promoted the formation of fibrils with R2 and R3 repeat units (Figure 3A). After 4 days of incubation, R2 fibrils and R3 protofibrils were formed. Furthermore, for R2 repeats production of ROS was pronounced, whereas R3 repeats were more prone to aggregation. The oligomerization was also observed after incubation with hydrogen peroxide, but the aggregation was more prominent in the presence of Cu^2+^ and was not prevented by high amounts of ascorbate, an endogenous antioxidant (Ahmadi et al., 2019). In the same study, glutathione, another important antioxidant from neuronal cells, did not prevent aggregation of pseudorepeats from microtubule-binding domains under limited access to oxygen. When repeat units were incubated with Cu^2+^ and GSH under ambient oxygen, more and larger protofibrils were formed for R3, which indicates an important role of GSH in R3 aggregation when Cu^2+^ is present (Ahmadi et al., 2019). Taken together, these findings demonstrate that the Cu^2+^-induced conformational changes and folds in tertiary structure may be viewed as a seed for dimerization and fibril formation afterward.

Shin and Saxena (2011) analyzed Cu(II) coordination of each of the octadecapeptides from the R1–R4 pseudorepeats. They suggested a square-planar Cu(II)-coordination geometry with three nitrogen donors and one oxygen donor. Carbonyl oxygen of a backbone amide and a nitrogen atom of the histidine imidazole ring probably directly participate in Cu(II) coordination in octadecapeptide fragments derived from all four repeat units (Shin and Saxena, 2011). Other studies also suggested that octadecapeptides may bind copper and emphasized the critical role of the histidine imidazole group in Cu(II) coordination (Ma et al., 2005; Zhou et al., 2007; Ahmadi et al., 2019). Of note, it seems that residues involved in interactions between microtubules and tau are not involved in copper binding. This suggests that copper can bind to microtubule-bound tau and affect the secondary structure of many tau molecules (Soragni et al., 2008).

As several studies indicate the important role of the R3 repeat on tau aggregation, smaller peptide sequences derived from the R3 unit were of particular interest when considering the effect of copper on tau aggregation (Ma et al., 2005). At least for the octadecapeptide ^318^VTSKCGSLGNIHHKPGGG^335^ derived from the R3 repeat of the longest tau isoform, copper-binding is very strong and of high affinity. In the study of Ma et al. (2005), imidazole rings of the two histidine residues from this octadecapeptide are critically involved in Cu^2+^ coordination, whereas no evidence was found that backbone amide or side chains of other amino acids participate in copper coordination at physiological pH. They suggested that octadecapeptide from the R3 repeat coordinates Cu^2+^ *via* the two imidazole rings of histidine residues, C-terminal carboxylic group, and N-terminal amino group in a square-planar geometry (Ma et al., 2005). Various factors may affect binding properties of copper to peptides derived from the R3 repeat. For the tau318–335 peptide, coordinating ligands and conformation are highly dependent on pH. At acidic pH, confirmation of the main chain in aqueous solution adopted a mixture of random structure, α-helices, and β-turns, whereas at physiological pH content of α-helices was increased (Ma et al., 2005). After adding 0.25 mol eq of Cu^2+^, the R3 peptide showed a monomeric α-helical structure, and the β-sheet was present after adding 1 mol eq of Cu^2+^ (Ma et al., 2005). It is commonly accepted that the β-sheet structure is essential for aberrant protein aggregation. However, α-helices also may trigger the formation of protein aggregates, and in addition to the β-sheet structure, α-helices are also found in PHFs, even though helix-breaking amino acids are abundant in tau protein (Sadqi et al., 2002). Therefore, these results suggest that the R3-derived 18-mer can initiate self-aggregation in the presence of very low concentrations of Cu^2+^. As α-helices and β-sheet turns are required for PHF formation, copper is likely involved in both the initial steps of self-assembly and formation of PHF-like structures afterward at later stages (Ma et al., 2005).

Because R2 repeat can also form filamentous polymers, copper-binding properties of the octadecapeptide ^287^VQSKCGSKDNIKHVPGGG^304^ from the R2 were also examined. The 18-mer derived from the R2 repeat binds copper with 1:1 stoichiometry and low affinity. At physiological pH, cysteine and the imidazole group of histidine are critical for copper coordination independent of the main chain. Cu^2+^ triggered the formation of α-helices in R2 fragment, and with increasing copper concentration, more α-helices were formed. The R2 peptide had a higher ability to form α-helices compared to the R3 peptide but aggregated at higher concentrations of copper (Ma et al., 2006). Similarly, peptide ^256^VKSKIGSTENLKHQPGGG^273^ from the R1 repeat may bind copper with 1:1 stoichiometry. The histidine residue is the main anchor for copper, but only minor changes in the secondary structure are induced upon copper-binding (Zhou et al., 2007). Interestingly, the aggregation of the octadecapeptide from the R1 repeat was inhibited in the presence of copper indicating that copper binding also could have an inhibitory effect on aggregation (Zhou et al., 2007). It is assumed that copper-stimulated aggregation of the R3 peptide could be dependent on the number of histidine residues in the sequence. Whereas the R3 peptide contains two histidine residues, only one is present in R1 (Ma et al., 2005; Zhou et al., 2007). Cu^2+^-induced structural changes in Cu-R4 complex were smaller in comparison to the Cu-R1 complex (Ahmadi et al., 2019). Ahmadi et al. (2019) have observed that both R1 and R4 repeats form adducts with one or two copper ions but found no evidence for copper complexation with R2 and R3 monomers. Instead, their results suggested that only R2 and R3 dimers form complexes with copper, indicating an important role of dimerization on Cu^2+^ complexation. In R1 and R4 repeats, it seems that Cu^2+^ interacts with the imidazole group of histidine, but dimerization was not observed probably because these repeats do not contain cysteine in their primary sequence. In cysteine-containing R2 and R3 repeats, histidine was involved in the interaction, but Cys residues were probably more involved in aggregation because in presence of Cu^2+^ their thiol groups were prone to disulfide bond formation. It is particularly interesting that the interactions of R2 and R3 repeats with Cu^2+^ resulted ROS generation (Ahmadi et al., 2019). Due to the redox activity of Cu^2+^, a disulfide bond is formed between the thiol groups of Cys residues (Figure 3B). This redox reaction also generates Cu^+^ ions that further catalyze ROS production *via* Fenton-type reactions (Ahmadi et al., 2019). Another study showed that Cu(II)-2His complex binds to cysteine, and during cysteine oxidation, a Cu(I)-2His complex is formed. Similarly, this complex promoted Fenton chemistry and an increase in ROS production. In a vicious cycle, the Cu(II)-2His complex was regenerated and was again capable to oxidize cysteine (Zåbek-Adamska et al., 2013). For R1 and R4 pseudorepeats, neither oligomerization nor ROS production was noticed (Ahmadi et al., 2019). The disulfide bond formation also may be initiated in the presence of hydrogen peroxide. However, the dimerization of R2 and R3 was more prominent in the presence of Cu^2+^ (Ahmadi et al., 2019). After examining the ability of ascorbate to reduce Cu^2+^ in the presence of all repeat units (R1–R4), it was found that R2 and R3 have a higher affinity for Cu^2+^ than ascorbate, implying that ascorbate cannot prevent R2 and R3 dimerization. Taken together, these findings suggest an important catalytic role of Cu^2+^ in oligomerization *via* disulfide bond formation and in ROS generation when R2 and R3 repeats are engaged in copper coordination.

## Copper Binding to Larger Tau Fragments

With regards to larger tau fragments, like the 198-residue K32 fragment that contains the four pseudorepeat motifs and the two flanking domains from the human tau, it was observed that in oxidative conditions, peptides ^287^VQSKCGS^293^ and ^310^YKPVDLSKVTSKCGS^324^ from K32 C-terminal participate in Cu(II) coordination. To a smaller degree, a tetrapeptide ^306^VQIV^309^, and several histidine residues (H299, H329, H330, H374, H388) were also involved in the interaction (Soragni et al., 2008). For the K32 fragment, only a small amount of aggregates were formed following copper binding due to the very slow rate of aggregation in physiological conditions, although a propensity toward β-structure was observed upon copper-binding (Soragni et al., 2008). For both full-length tau and K32 fragment, Soragni et al. (2008) found only one binding site for Cu^2+^ per monomer, with critical residues mainly located in repeats R2 and R3. It seems that copper simultaneously binds to R2 and R3 repeats, bringing reactive groups into spatial proximity. Within R2 and R3 sequences, two cysteine residues (C291 and C322) are identified as essential for the binding of copper to tau. Given the copper-induced oxidation of these cysteines, the intramolecular disulfide bridge between their thiol groups is probably formed as a result of copper:tau interactions (Soragni et al., 2008). Hence, this study demonstrates copper-promoting effects on tau aggregation confirming the important role of cysteine residues from the R2 and R3 repeats in the process of oligomerization.

Copper coordination was also examined with peptides derived from the N-terminal part of tau, outside the microtubule-binding domain. Two tau fragments, containing residues 1–25 or 26–44, can anchor copper *via* two putative binding sites, the imidazole side chain of the histidine residue or the N-terminal amino group of the peptide chain. Conformations of both peptides were changed after the formation of Cu(II) complexes. Interestingly, both peptide fragments inhibited Cu(II)-mediated aggregation of Aβ, and the effect was more pronounced for tau26–44 (Di Natale et al., 2018). Fragments of tau protein originating from the N-terminus, including tau26–44 peptide, are detected in CSF and brain tissue from the AD patients (Barthélemy et al., 2016; Zhou et al., 2018). Fragment tau26–44 in physiological conditions is not prone to aggregation due to intrinsically disordered structure, but it represents the minimal amino acid sequence capable of inducing NMDA receptor-mediated neuronal death (Amadoro et al., 2006). Chronic exposure of neuronal cultures to tau26–44 fragment resulted in many features characteristic of presymptomatic stages of AD pathology such as neuritic dystrophy, breakdown of microtubules, loss of mitochondria and impairment of oxidative phosphorylation, indicating that the extracellularly secreted N-terminal truncated tau fragments may largely contribute to the development of AD pathology (Atlante et al., 2008; Florenzano et al., 2017).

The effects of copper were also studied in animal models that mimic tau pathology (Sedjahtera et al., 2018; Ishihara et al., 2019). For example, in 3XTg-AD mice, chronic exposure to copper in drinking water exacerbates tau hyperphosphorylation and upregulates cdk5 activator p25, whereas the activity of GSK-3β remains stable. Hyperphosphorylation of tau was detected at Ser396/Ser404 (PHF-1) epitope, indicating the presence of late-stage pathological tau and tangle formation (Kitazawa et al., 2009).

The impact of tau acetylation on tau’s ability to bind copper ions and the effect of copper ions on the aggregation of acetylated tau is largely unknown. It was recently shown that the acetyl mimicking mutation K274Q (lysine mutated to glutamine) enhances the binding affinity for Cu^2+^, and Cu^2+^ ions more strongly promote aggregation of K274Q when compared to normal tau (Rane et al., 2020). This suggests that acetylation additionally increases Cu-induced toxicity and contributes to neuropathological processes (Rane et al., 2020). The effect of copper on tau glycosylation yet needs to be determined. For some proteins, it has been shown that their glycated forms are more prone to copper-mediated oxidation (Kobayashi et al., 1995), at least in part *via* a mechanism that involves the production of free radicals and protein cross-linking (Chace et al., 1991). Based on these findings, it could be expected that copper will also be capable of promoting aggregation of glycosylated tau forms.

Finally, the specific contribution of copper in glial tauopathy remains to be determined. OS-related markers have been observed in tauopathy of glial cells (Kahlson and Colodner, 2016), and toxic insult that increases the production of ROS results in tau cleavage and formation of NFTs in C6 rat astroglioma cell line (Means et al., 2017). Further studies are needed to examine the effects of copper deregulation on glial tauopathy. Based on the important neuron-supportive role of glial cells, a better understanding of the OS-driven mechanisms involved in glial tau aggregation could be critical for the identification of novel targets for effective therapies in AD and other tauopathies.

## Targeting Copper Levels as a Potential Therapeutic Approach in AD

Overall these findings indicate that natural or synthetic copper chelators could be considered as potential therapeutic agents in AD. In general, metal-targeted therapy is aimed at metal removal or redistribution. Chelators are intended to disrupt interactions with metal ions and specific molecules, ultimately promoting their excretion and preventing detrimental consequences (Esmieu et al., 2019). However, chelation therapy has achieved controversial clinical results. For effective and safe prolonged chelation therapy, the time of application and dosage of chelating agents must be carefully optimized (Hegde et al., 2009). The selectivity of metal chelation is also essential due to the risk of general metal depletion and interference with metal balance. As an increase in divalent cations is characteristic for the early phase of AD, potential copper chelators are expected to be beneficial only for patients in the early phase of the disease. Taken together, the development of safe copper chelators intended for prolonged use, and directed exclusively to the removal of copper-containing deposits in AD, is very challenging. Nevertheless, targeting copper levels still represents a promising option in AD, particularly considering that increased levels of loosely bound copper are a characteristic finding in the serum of AD patients (Bucossi et al., 2011).

Considering the multifactorial etiology of AD and numerous mechanisms contributing to the disease development and progression, effective therapeutic approach may rely on multi-target drugs (Sharma et al., 2018). As various factors govern tau pathology, copper-targeted approaches in alleviating progression of the disease probably would be more useful if combined with some other interventions targeting tau pathologies, such as inhibition of tau kinases, inhibition of post-translational tau modifications and tau aggregation, active and passive immunotherapy and modulation of tau degradation processes, among others. In that regard, the neuroprotective potential of various polyphenolic compounds, such as flavonoids, has been evaluated. These dietary antioxidants exert multifunctional effects, i.e., they act as metal chelators, ROS scavengers, and modulators of redox signaling (Jazvinšćak Jembrek et al., 2012; Ayaz et al., 2019).

Quercetin is one of the most studied and the most potent scavengers of ROS (Jazvinšćak Jembrek et al., 2012, 2014). It prevents *in vitro* fibrillization of recombinant tau protein (2N4R isoform; Kumar et al., 2019). Molecular dynamic simulations of the tau fragment containing the R2 domain and the ^306^VQIVYK^311^ hexapeptide motif have revealed conformational changes upon interaction with quercetin. Quercetin interacts with tau protein in the close vicinity of the most aggregation-prone region and stabilizes the native random coiled state of the monomeric tau by specific hydrogen bonding and hydrophobic interactions (Kumar et al., 2019). Furthermore, quercetin was effective against okadaic acid-induced tau hyperphosphorylation in HT22 mouse hippocampal cells by inhibiting cdk5 activity (Shen et al., 2018). Chelation of quercetin to copper may inhibit the formation of hydroxyl radicals and exhibit a protective effect when quercetin is present in excess over copper (Jomova et al., 2017). However, it should be kept in mind that in the presence of copper ions quercetin may exhibit both antioxidative and prooxidative activities, and the outcome of copper/quercetin interactions is not straightforward (Filipe et al., 2004; Zubčić et al., 2020).

Another examined flavonoid is epigallocatechin-3-gallate (EGCG) from green tea. Oral administration of EGCG in drinking water in mice with the Swedish *APP* mutation suppressed the formation of sarkosyl-soluble phosphorylated tau isoforms and improved cognitive performance (Rezai-Zadeh et al., 2008). In rat primary neurons EGCG increased the clearance of phosphorylated tau species (Chesser et al., 2016) and inhibited the aggregation of tau fragment His-K18ΔK280 into insoluble, high-molecular-weight oligomers at substoichiometric concentrations. This fragment spans the microtubule-binding domain of the longest human tau isoform but lacks Lys280, and readily adopts β-sheet structures resulting in the formation of toxic and oligomeric tau aggregates. EGCG may exert its effects by preventing conformational changes by specifically interacting with early aggregation intermediates and preventing their seeding activity (Wobst et al., 2015). EGCG acts as a ROS scavenger and prevents OS-induced DNA damage and Aβ-induced lipid peroxidation, and increases the production of GSH by activating the Nrf2 antioxidant pathway (Ayaz et al., 2019). Moreover, EGCG may interfere with the Cu(II)-induced fibrillization and aggregation of α-synuclein, whose abnormal aggregation is characteristic of Parkinson’s disease and reduces the generation of Cu(II)-induced production of ROS (Teng et al., 2019).

Curcumin, an extract from the rhizome of the plant *Curcuma longa*, is highly recommended as a health-promoting polyphenolic nutraceutical. As a multi-target compound, it is also considered for the prevention and treatment of AD. It readily crosses the blood-brain barrier, preserves synaptic structure and functions, and positively affects learning and memory abilities in AD rats (Reddy et al., 2018). Curcumin has powerful antioxidant properties. It reduces OS acting as an effective ROS scavenger, stimulates the activity of superoxide dismutase and catalase, elevates glutathione level, and decreases lipid peroxidation (Chen et al., 2018). As a natural β-diketone ligand, curcumin strongly chelates various metals, including copper. Accordingly, it is considered that curcumin could be effective against copper-induced neurotoxicity in AD (Wanninger et al., 2015). In N2a/APP695swe cells and APP/PS1 transgenic mice curcumin reduced tau phosphorylation and activity of GSK-3β (Sun et al., 2017). Similarly, in Tg2576 mice overexpressing Aβ, the administration of turmeric extract for 6 months reduced the level of hyperphosphorylated tau by ~80% (Shytle et al., 2012). *In vitro* studies have shown that curcumin binds to 4R0N adult tau, prevents the formation of β-sheet structure and inhibits tau oligomerization and filamentation, and is capable to induce disaggregation of the preformed tau oligomers and fibrils (Rane et al., 2017). Based on these three examples, it is likely that natural compounds, acting as potent metal chelators and antioxidants, could be a promising approach in the prevention and therapy of Cu-related tau pathology. Consequently, further studies are needed to explore and better explain the potential beneficial effects of natural polyphenolic compounds against copper-induced toxicity in animal models and clinical studies.

Small synthetic ligands acting as copper chelators have already been designed, primarily to remove copper from Aβ aggregates. As mentioned previously, chelating molecules must have some properties. They have to be nontoxic, capable to cross the blood-brain barrier and bind copper with moderate affinity. It is also important for chelating agents to chelate unbound copper ions and sink copper ions from the deposits, but not to remove metal ions from various metalloproteins (Sharma et al., 2018). Probably the most studied copper-chelating agents are clioquinol derivatives that are efficient in removing metal ions from Aβ and disassembling of preformed Aβ aggregates. They can improve cognitive functions in a transgenic animal, but the evidence of their positive effects in clinical studies is still missing (Budimir, 2011; Sharma et al., 2018; Esmieu et al., 2019). Regarding copper complexation and tau pathology, few studies have been performed. In human neuroblastoma cells incubated with excess copper for 24 h, the increase in tau phosphorylation at AD-specific sites was reduced by a copper complexing agent that decreased expression of the p35/p25 activators of cdk5 (Voss et al., 2014). In the same study, oral administration of zinc acetate in drinking water depleted environmental brain copper levels in male mice expressing wild-type human tau (hTau strain). Zinc acetate increased the overall expression of tau protein and reduced tau phosphorylation at Ser202/Thr205, Thr231, and Ser396/Ser404 (PHF-1 epitope). Expression of p35/p25 activators and cdk5 was unchanged, whereas GSK-3β and its inhibited form phosphorylated at Ser9 were depleted, probably contributing to decreased tau phosphorylation when copper levels were reduced (Voss et al., 2014). However, these changes in expression level and phosphorylation patterns did not improve spatial learning and memory of 18-months-old hTau animals (Voss et al., 2014).

In another study, it was shown that benzothiazole-containing compounds can bind Cu^2+^ and form complexes with a prominent free-radical scavenging activity. Theoretically, by competing with tau for Cu binding, these compounds may reduce Cu-induced damage in the brain (Geng et al., 2012). Bis(8-aminoquinoline) ligands are yet another example of specific and efficient Cu^2+^ chelators that may remove Cu^2+^ from amyloids and attenuate ROS production. In a reduced environment, they are demetallated and release copper ions that may help in regaining copper homeostasis under reductive conditions (Nguyen et al., 2014). Finally, an interesting study was performed with a GSK-3β inhibitor termed Cu^II^ (gtsm). The study indicated that delivering more copper can indirectly exert a beneficial effect on tau pathology. Cu^II^ (gtsm) is a metal complex that delivers Cu into cells and increases the intracellular bioavailability of copper. When exposed to a reducing environment, Cu^II^ is reduced to Cu^I^ and dissociates from the ligand, thus increasing levels of copper inside the cell (Crouch et al., 2009). In SH-SY5Y cells, Cu^II^ (gtsm) inhibited the activity of GSK-3β and decreased tau phosphorylation at Ser404 and Ser396. By comparing effects of Cu^II^ (gtsm) and negative control, namely, compound Cu^II^ (atsm) that is not prone to reduction and dissociation, it was found that increased bioavailability achieved by Cu^II^ (gtsm) is required for the reduced activity of GSK-3β (Crouch et al., 2009).

By searching for AD-modifying therapy, a better understanding of metal dyshomeostasis and the exact potential of antioxidant/chelating strategies in slowing down tau-related pathology must be further explored, particularly in preclinical and clinical studies. Despite some promising findings, the relevance of targeting copper levels for the improvement or reversal of pathophysiological changes in AD is still controversial. Due to aberrant copper homeostasis in AD, the brain metal redistribution and re-establishing of metal homeostasis, rather than metal removal, could be considered as a primary goal of metal targeted strategies (Hegde et al., 2009). It is crucial for future studies to deduce the protective potential of copper-based strategies and to better understand the effects of these potential approaches on the progression of tau pathology. More pharmacological and toxicological studies are needed to better understand the pharmacodynamic and pharmacokinetic properties of these potential copper-modifying agents. In that regard, the aforementioned natural compounds, applied either alone or in a combination with some other drugs, could be a promising approach based on their general safety and the ability to attenuate several copper-related effects.

## Conclusion

Deregulation of copper homeostasis promotes an OS condition that is recognized as an early event in AD. Copper-tau interactions represent an important molecular factor contributing to the pathological changes in AD. Recent findings demonstrate the catalytic role of Cu^2+^ in aggregation as it triggers conformational changes of tau peptides located in the microtubule-binding domain. Cu^2+^ not only promotes aggregation but contributes to increased ROS formation, further promoting OS conditions and neuronal damage. Metal ion chelators and antioxidants capable to reduce copper-mediated oxidative injury and propagation of tau aggregation have been suggested as promising approaches in alleviating AD progression. Natural polyphenolic compounds have metal-chelating and antioxidative properties that make them promising and safe multi-target drug candidates. Paradoxically, there are reports which suggest that delivering more copper, instead of less, might be beneficial. Further investigation of the interactions between tau and copper might help in the development of novel copper-chelating approaches that hopefully will modify the progression of the disease after regaining copper homeostasis. Based on many pathological mechanisms contributing to AD progression, and taking into consideration that chelating approaches must be applied with caution as chelators are not selective and levels of copper and other metals should not be over-reduced, a combined therapy directed against multiple processes is expected to yield better efficacy. In that regard, inhibitors of tau aggregation as well as dissociation agents (such as immunotherapy), could be applied together with chelators and antioxidative agents.

## Author Contributions

MJ wrote the initial draft. KZ contributed to literature collection. PH and GŠ supervised and edited the manuscript. All authors contributed to the article and approved the submitted version.

## Conflict of Interest

The authors declare that the research was conducted in the absence of any commercial or financial relationships that could be construed as a potential conflict of interest.

## Abbreviations

AD: Alzheimer’s disease
Aβ: amyloid-β peptides
cdk5: cyclin-dependent kinase 5
ERK1/2: extracellular signal regulated kinase-1/2
GSK-3β: glycogen synthase kinase-3β
I_2_^PP2A^: inhibitor-2 of protein phosphatase-2A
MAP: microtubule-associated protein
MAPKs: mitogen activated protein kinases
NFT: neurofibrillary tangles
OS: oxidative stress
PHF: paired helical filaments
PP2A: protein phosphatase 2A
ROS: reactive oxygen species
RNS: reactive nitrogen species.
